# Smartphone-based low light detection for bioluminescence application

**DOI:** 10.1038/srep40203

**Published:** 2017-01-09

**Authors:** Huisung Kim, Youngkee Jung, Iyll-Joon Doh, Roxana Andrea Lozano-Mahecha, Bruce Applegate, Euiwon Bae

**Affiliations:** 1 Applied Optics Laboratory, School of Mechanical Engineering, Purdue University, West Lafayette, Indiana 47907, USA; 2 Universidad Nacional Colombia-Palmira, Palmira Colombia; 3 Department of Food Science, Department of Biological Sciences, Purdue University, West Lafayette, Indiana 47907, USA

## Abstract

We report a smartphone-based device and associated imaging-processing algorithm to maximize the sensitivity of standard smartphone cameras, that can detect the presence of single-digit pW of radiant flux intensity. The proposed hardware and software, called bioluminescent-based analyte quantitation by **s**martphone (BAQS), provides an opportunity for onsite analysis and quantitation of luminescent signals from biological and non-biological sensing elements which emit photons in response to an analyte. A simple cradle that houses the smartphone, sample tube, and collection lens supports the measuring platform, while noise reduction by ensemble averaging simultaneously lowers the background and enhances the signal from emitted photons. Five different types of smartphones, both Android and iOS devices, were tested, and the top two candidates were used to evaluate luminescence from the bioluminescent reporter *Pseudomonas fluorescens* M3A. The best results were achieved by OnePlus One (android), which was able to detect luminescence from ~10^6^ CFU/mL of the bio-reporter, which corresponds to ~10^7^ photons/s with 180 seconds of integration time.

Luminescence based detection methods have been used in biology, chemistry, and the medical field due to their unique characteristic of self-photon generation from chemical energy. Among these methods, bioluminescence is extremely attractive as the genetics and biochemistry are known for many luminescent organisms. The genes from these systems can be cloned and expressed in bacteria. The expression of these genes can also be integrated with genetic regulatory elements to sense physical and chemical changes in the bacteria’s environment and produce a luminescent response. These luminescent reporter bacteria can be interfaced with optical transducers resulting in biosensors for numerous monitoring applications as well as reagents in the application reported in this work.

The recent trends of integrating everything into network connectivity such as the internet of things (IoT) have drawn interest from numerous areas of research. At the core of this new trend, the smartphone becomes a versatile platform with tremendous potential for scientific instrumentation. Owing to their inherent nature, smartphones have 1) high computing power, 2) high-resolution complementary metal-oxide semiconductor (CMOS) sensors, and 3) network and geotagging capability. In addition, compared to other laboratory equipment, smartphones are inexpensive and can be easily converted to portable instruments with appropriate accessories. Numerous smartphone-based analytical devices have been previously reported including: spectrometers^1^,^2^, microscopes^1^,^3^,^4^,^5^, fluorimeters^6^,^7^,^8^,^9^, colorimetric devices^10^,^11^,^12^, and instruments for immunochemistry^13^ and microbiology^14^. Luminescence detection by mobile phones include a bio-luminescence assay to detect bile acid and chemo-luminescence assays for cholesterol detection^15^, lactate in oral fluid^16^, and salivary cortisol level^17^. Most of these chemo- and bioluminescence based assays utilize the lateral flow strip as their test substrate and report only relative comparisons of light intensities for the detection limit of the analyte concentration. The qualitative representations of light intensities make it difficult to compare performance across different modalities and instruments.

The standard photo-detectors for bioluminescent measurements are photomultiplier tubes (PMT), the photo-electron multiplication effect of the PMT allows detection of extremely low levels of luminescence from biological samples. Most report the radiant flux of bioluminescence detection is in the range of 10^4^–10^7^ photons/s, which is enough for PMTs. However, PMTs require special high-voltage circuitry for their operation, making them more expensive than other alternative detectors. They are also susceptible to magnetic fields, and can potentially be damaged by overexposure. Avalanche photodiodes (APD) are an alternative for luminometry but they still require special circuitry to realize the breakdown phenomena. In this paper, we have explored the feasibility of utilizing default CMOS sensors in smartphones, taking advantage of recent advances in smartphone electronics such as back-illumination CMOS technology and significantly improved photon sensitivity of the detectors^18^,^19^. We also take advantage of the improved software to apply an image-processing algorithm to effectively remove random noise, thus increasing the signal-to-noise ratio for detection of ultra-low luminescence. To facilitate ease of use, light sequestration and efficient photon collection a sample holder was designed and manufactured using 3D printing. Recent operating systems are allowing users to control the exposure time of the smartphone camera which will decrease detection limits and allow increased dynamic range.

Here we report an integrated bioluminescence readout system consisting of a simple, portable, and low-cost sample holder with associated algorithms, that effectively translate luminescence intensity to concentration for a bacterial sample. The 3-D printed holder provides a light-tight environment and positions the sample at the same location. As a proof of concept, we used bioluminescent reporter bacteria that emits photons at 490 nm to determine the detection limits of light intensity measurements from different smartphones.

## Results

### LED-based calibration

As shown in Table 1, 9 different OD values were generated by utilizing combinations of calibrated ND filter sets. Estimated attenuated power, *P*_in_, passing through the filter set is calculated by


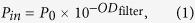


where *P*_0_ is the nominal input power without any ND filters, and *OD*_filter_ is the effective optical density value from the combinations of ND filters. Since many bioluminescence applications report their work in terms of photon counts per second (cps)^20^, we adopt this quantity as


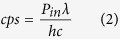


where *h c*, and λ is the Plank constant, the speed of light, and wavelength respectively. The result indicates that with OD value of 4–8 we can generate a cps range of 10^8^ to 10^4^, which is within the range of typical bioluminescence measurements.

### Effect of the NREA algorithm

**Figure 1:**
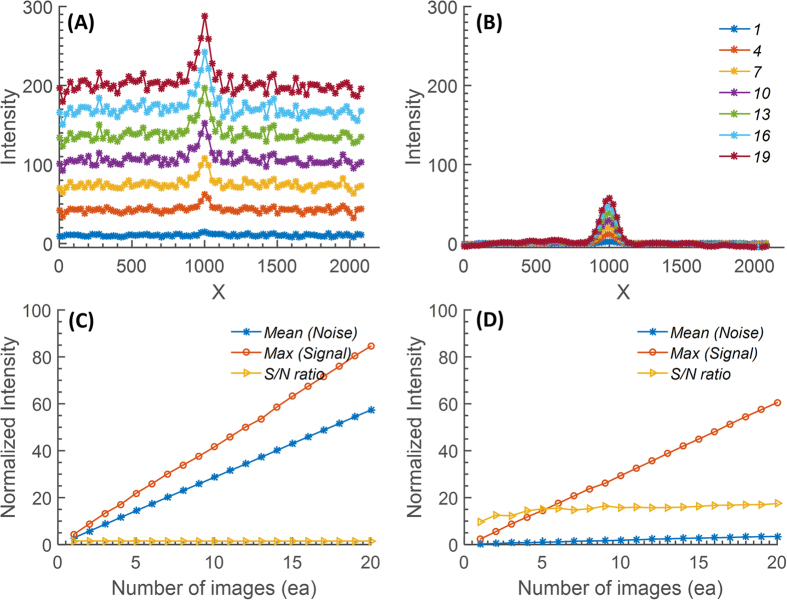
Comparison of (**A**) normal (no algorithm applied) and (**B**) NREA-applied accumulation results. A total accumulation of 20 images was made for the comparison, and a cross-sectional view at the LED center area from every three results is shown. The S/N ratio comparison along the number of accumulated image is shown at (**C**) normal accumulation, and (**D**) NREA applied accumulation. Mean value of the noise, maximum of the signal (peak intensity value at the LED center area), and computed S/N ratio are visualized. Both the slope and the value of the signal from the normal accumulation is higher than that of the NREA-applied case; however, the mean of the noise is also higher. So, overall the S/N ratio for the normal accumulation case is worse than that of the NREA-applied case.

**Figure 2:**
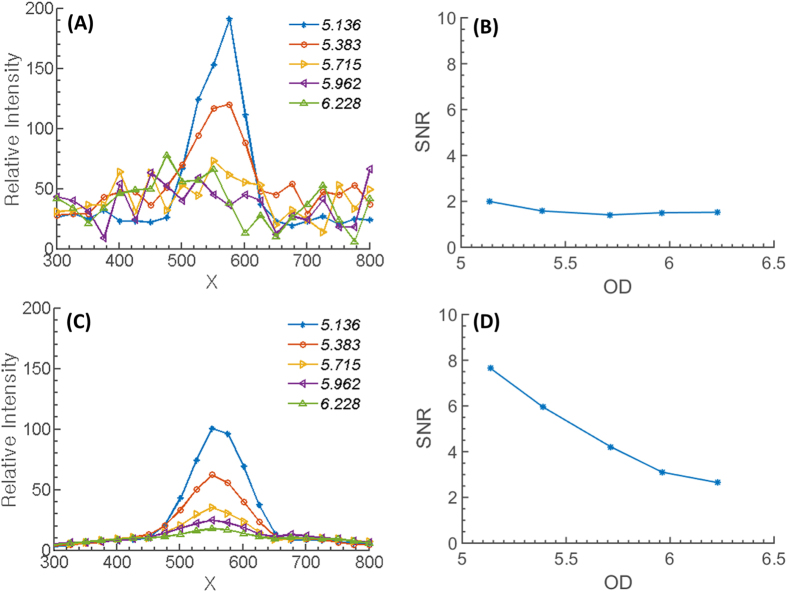
Comparison of before and after applying the NREA algorithm. (**A**) 1-D cross-section of the intensity versus the OD values for simple accumulation of a series of low-light LED images. (**B**) The associated SNR. (**C**) 1-D cross-section of the intensity versus the OD values using the NREA algorithm. (**D**) Trend of the SNR when noise level was calculated for the average intensity of the background region.

As shown in formulation of the noise minimization algorithm section, the mathematical description of the noise-cancelation algorithm is effective when the input signal level is close to the noise level. Figure 1 displays the effect of the proposed NREA algorithm for low light detection. For the comparison, a simple accumulation algorithm is applied to the series of low-light images and their SNRs are plotted (Fig. 1(C)). Performance does not improve since both noise and signal are accumulated. Meanwhile, the NREA algorithm effectively reduces the noise while preserving the desired signal. Therefore, SNR increases up to integrating five or more images at a given filter OD and plateaus after that (Fig. 1(D)). For a more detailed analysis, five intermediate OD values are measured between OD5.136 and OD6.228. Figure 2(A,B) shows the 1-D cross-sectional intensity profile for the simple accumulation algorithm; the associated SNR shows a plateau below OD5.718 where the signal and the noise becomes indistinguishable. Meanwhile, when the NREA algorithm is applied, the absolute value of the maximum intensity is lower than the simple accumulation algorithm; the significant reduction of the inherent noise level improves the overall SNR up to four times that of the simple accumulation algorithm (Fig. 2(C,D)). NREA algorithm is developed utilizing Matlab, and all of the analyses are done at PC environment (Intel i5 750 (2.67 Ghz), 8 Gb RAM). The analysis time for five accumulations with NREA takes 0.15 seconds for 480 × 640 pixel image set.

### Effect of optical chamber

**Figure 3:**
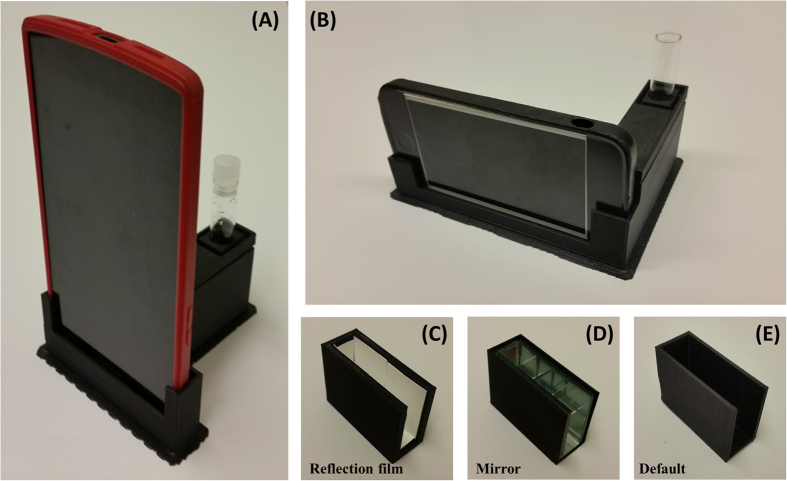
Detector chamber for BAQS. (**A**,**B**) Shows BAQS for two different models. (**C**,**D**), and (**E**) displays a reflection film module, a mirror surface module, and default sample chamber, respectively.

A standard method of bioluminescence detection utilizes a 12 × 75 mm glass tube for the measurement. Therefore, to compare with the reference instrument, we have fabricated a sample chamber that can hold the same glass tube. Since the light intensity is extremely low, efficient capture of photons is important. The luminescent light radiates through the curved surface of the glass tube, and as a result the camera of the smartphone captures only approximately half of this light. To maximize the efficiency of capturing the radially emitted photons, three different types reflectors – a diffusive reflection polymer film (diffusive reflection), a 4- to 6-λ first-surface mirror (specular reflection), and ABS material (default), are implemented in the chamber as in Fig. 3(C–E). In each case the green LED is located at the center of the chamber as a light source, and the output intensities of each material are measured with a power meter to quantify the enhancement of photon-capturing efficiencies. To avoid sensor saturation, ND filters are placed in front of the probe. The overall output intensities of each design were measured as 678 nW (diffusive film), 200 nW (mirror), and 46 nW (ABS) respectively by the power meter. The overall output signal was also measured with a smartphone camera (LG G2). Utilizing the NREA algorithm, the output intensities of each design are shown in Figure S4(A). As the result shows, the diffusive film enhances not only the maximum intensity (up to three-fold), but also the illuminated area (up to three-fold), while the first-surface mirror slightly enhances the maximum intensity and the area. To verify the effect of each material along the different input intensity for the lower detection limits, five different ND filter combinations were utilized; the results are shown in Figure S4(B), where RLU/pixel was calculated by dividing the sum of intensity by the pixel area above the threshold (intensity > 0). As indicated by the power-meter measurement, the diffusive film provided the best overall performance; however, the enhancement diminished as the incoming light level decreased.

### Inter-phone performance

**Figure 4:**
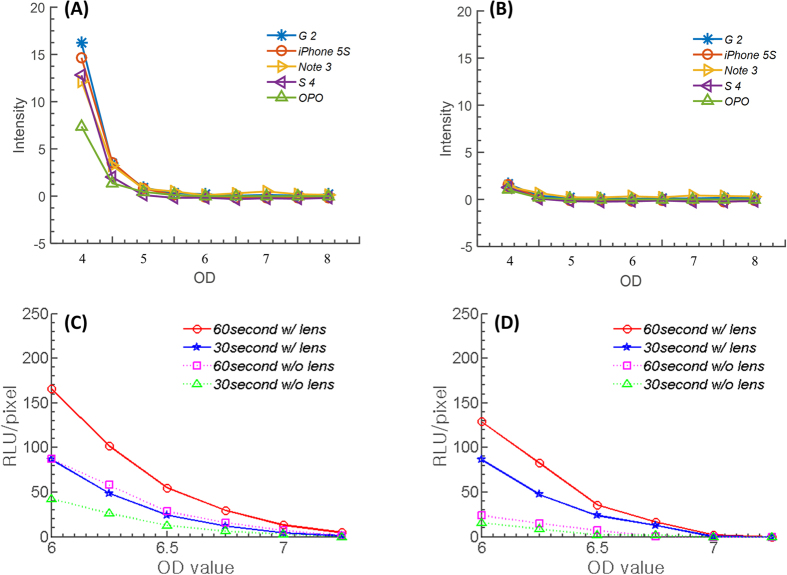
Standard and maximum-performance test for inter-phone variations. (**A**) Standard test for 5 different smartphones with ND filters providing OD4 to OD9 input-light power reduction with a *f* = 25 mm lens. iPhone 5 s and LG G2 provided the best performance on the standard performance test. Intensity profile plateaus below OD5 or 5.5. (**B**) The same result without the focusing lens. For maximum-performance tests, iphone 5 s and Oneplus One were selected. (**C**) Maximum performance test for Oneplus One. (**D**) Result for iPhone 5 s, where a maximum performance of OD6-6.5 is possible. Utilizing 60 seconds of exposure time with commercial apps and NREA simultaneously, both phones were able to detect the presence of light intensity down to OD6.5 to 7.

Figure 4 show the inter-phone performance comparison. Galaxy S4 (Samsung Electronics, Seoul, Korea), Galaxy Note 3 (Samsung Electronics, Seoul, Korea), LG G2 (LG Electronics, Seoul, Korea), OPO (Oneplus, Shenzhen, China), and iPhone 5 S (Apple, Cupertino, CA, USA) were selected for comparison. The comparison was performed in two steps: standardized test and maximum-performance test. For the standardized test, performance order was G2 > iPhone 5 S > Note 3 > S4 > OPO under the same experimental conditions. In addition, utilizing a plano-convex lens (*f* = 25 mm, diameter = 10 mm) dramatically increased performance up to 17 times in the lower OD regions. However, the limit of detection was close to OD5 for Android phones and OD5.5 for iPhone 5 S.

For the maximum-performance test, we chose iPhone 5 S and OPO since all the other phones limited exposure time to 1/6–1/5 sec^21^. We used the commercial app (FV5) with exposure settings of 15, 30, and 60 seconds for the OPO smartphone with exposure +2 level and the NREA algorithm. For the iPhone 5 S, we used a commercial long-exposure app (Manual –version 1.1.2) with 15, 30, and 60 seconds of exposure time and the NREA algorithm. Figure 4(C,D) shows the OD versus RLU/pixel trend. The result indicated that both handsets had a maximum low-light performance of OD 6.58, which corresponds to approximately 10^6^ photons/s, with the help of the optical chamber and NREA algorithm.

### Bioluminescence detection

**Figure 5:**
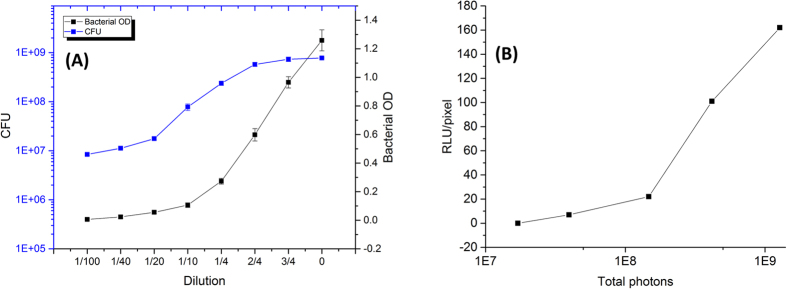
Bioluminescence detection from *P. fluoresecens* M3A strain. (**A**) Correlations between sample dilution, bacterial OD (600 nm), and CFU. Each data point is the mean of three replicate experiments. (**B**) Correlation between the estimated RLU/pixel and the total number of photons collected on BAQS. The system was set for the best performance setup (diffusive chamber, lens, integration time of 60 seconds, and 5 consecutive shots). For quantitative comparison, total photons were also expressed in terms of the filter OD of the neutral density filter combinations, based on cps*Δt*n, where Δt represents the integration time of each image and n is the number of images taken. Total number of photons were calculated with Δt = 60 sec and n = 3 images.

To verify the detection limit of the smartphone camera for bioluminescence detection, *P. fluorescens* M3A was used as a target organism. Figure 5(A) shows the relationship among bacterial OD, CFU, and dilution series, where bacterial OD_600_ of 1.25 correlates with a cell number of 7.8 × 10^8^ CFU/mL. Bacterial samples were measured by the reference luminometer; bacterial OD_600_ of 0.008 and 0.019 resulted in an average of 1.37 × 10^7^ and 1.83 × 10^7^ cps respectively, whereas higher OD values resulted in overload of the signal. Figure 5(B) shows the conversion data for RLU/pixel to the total number of photons collected on the BAQS. The system was set for the best performance setup (diffusive chamber, lens, integration time of 60 seconds, NREA with 3 consecutive shots), based on the cps*Δt*n, where Δt represents the integration time of each image, and n is the number of images taken. Total number of photons were calculated with Δt = 60 sec and n = 3 images.

**Figure 6:**
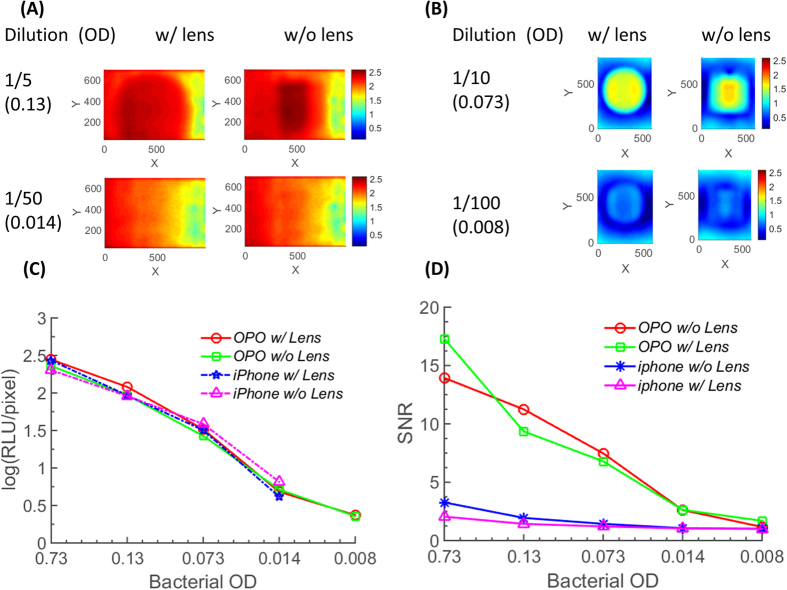
Bioluminescence detection from *P. fluoresecens* M3A strain with BAQS. (**A**) Representative luminescence image for 1/5 and 1/50 dilution sample for iphone 5 s. (**B**) Representative luminescence image for 1/10 and 1/100 dilution sample for OPO. Data were captured with and without lens (f = 25 mm, plano-convex) for comparison. Color scale bar is in logarithmic scale, and the best performance setup (diffusive chamber, lens, integration time of 60 seconds, 3 consecutive shots, and NREA algorithm) was used for data acquisition. (**C**) Estimated RLU/pixel for diluted bacterial samples for both phones. (**D**) SNR of the experimental data. Noise intensity was calculated by spatially filtering and averaging pixel intensity outside the signal area.

Figure 6 displays the result from the BAQS when two handsets (iPhone and OPO) were challenged with the bioluminescent bacterial sample. Figure 6(A) shows the image after processing with the NREA algorithm for bacterial OD_600_ of 0.13 and 0.014 with and without the lens (data plotted in log scale), while Fig. 6(B) shows the result from the OPO. Owing to the lower signal background from the CMOS sensor, the OPO was able to measure down to a bacterial OD of 0.008. For quantitative analysis, total signal and SNR within the region of interest were plotted in Fig. 6(C,D). The SNR of the OPO was clearly superior to that of the iPhone 5 S across all bacterial sample dilutions owing to the different background level from the sensor. The lower detection limit of the OPO is equivalent to approximately 7.9 × 10^6^ CFU/mL, while that of the iPhone 5 S is measured as approximately 2 × 10^8^ CFU/mL. From the reference luminometer, a bacterial OD of 0.008 resulted in 1.3 × 10^7^ cps, which corresponds to ~10^8^ total photons for an integration time of 8 seconds. For the BAQS, the same sample resulted in ~8 RLU/pixel, and this corresponds to ~8.82 × 10^7^ total number of photons over 180 seconds of integration time (Fig. 6(B)), which is similar to the luminometer output values. However, the number of photons captured per second for BAQS was only 4.9 × 10^5^, close to 2 orders of magnitude lower than that of the luminometer. From the experimental verification, the lowest detectable CFU for the current design of the smartphone-based bioluminescence measurement system is approximately 7.9 × 10^6^ CFU/mL.

## Discussion

The NREA algorithm is based on suppression of the random noise signal by ensemble averaging while preserving the additive nature of the desired signal. Compared to the typical additive algorithm, which increases both signal and noise together, NREA provides better performance, especially dealing with low levels of light intensity where the level of signal is similar to that of the inherent dark noise of the sensor. However, Fig. 1 shows that, even with NREA algorithm, overall SNR plateaus after five or more images have been accumulated, which suggests that no more than three to five consecutive images are needed in order to apply the NREA algorithm. This can be understood as the fundamental limit to the improvement that can be achieved even after suppressing the background noise level. Maximum SNR with approximately three to five images accumulated is beneficial for the actual bioluminescent imaging, since unlike the LED light source, bioluminescent light is a time- and diffusion-dependent process, which inherently shows a decaying intensity versus time. Considering the 15-, 30-, and 60-second integration time of each single bioluminescence image from *P. fluorescens* M3A samples, it takes 45–180 seconds to acquire the optimal number of images to process with the NREA algorithm. The merits of the NREA algorithm are applicable for other low-light optical measurements, such as smartphone-based luminescence technique^11^,^15^,^17^,^22^, fluorescence detection^2^,^7^,^8^, and spectrometry^2^,^8^ to name a few.

A few interesting points were found during this research. First, one critical limitation of the smartphone-based luminescence detection is that the inherent dark or leakage current from the CMOS sensor itself can deteriorate the performance of low-light detection. As shown in Fig. 6(A) and Figure S3, most commercial smartphones except recent OPO models, have an inherent dark signal, which does not affect typical photography but significantly reduces the SNR for the low-light conditions. Among the handsets tested, the only smartphone to show superior dark-current level was the OPO (Fig. 6(B,D)), and more recent phones will be expected to provide better low-light characteristics. Second, for the same camera settings (ISO, f-number, and shutter speed), the LG G2 has the best sensitivity in a medium (~OD 4–5) level of light. However, the manufacturer has blocked manual control of the shutter speed and limited the maximum speed to 0.006 to 0.5 seconds, whereas the iPhone 5 S and OPO allows app developers to control the shutter speed over a longer period time (up to 60 seconds). Therefore, even with less superior device specifications, the OPO provided the best resulst for low-light applications with the help of longer integration times and the NREA algorithm. Even though the OPO was found to be the best model for bioluminescence detection among the tested models, more handsets will have improved lower light sensitivity and manual control of the camera function in the near future. Therefore, we expect more smartphones will be able to detect the bioluminescence signal in near future. Third, we have to understand the inherent nature of the smartphone imaging lens and the associated focusing system. Most smartphones are designed for photography and include autofocusing mechanisms operated by the voice-coil motors. In an extreme low-light condition, the autofocusing mechanism seems to have difficulty in positioning the lens for the best focus. Current sample-chamber design employs reflection materials and a plano-convex lens for efficient capture and focusing of bioluminescence photons. One notable loss of signal occurs on the back side of the circular glass tube, which is not directly imaged by the smartphone camera (Fig. 3(A,B), Figure S2(B)). If we assume that all the backward-directed luminescence photons were redirected to the camera for imaging, that would potentially double the intensity level and lower the detection limit of the bacterial OD. This will require the design of a parabolic mirror or integrating sphere that can direct the photons to the front-imaging plane.

Applications of luminescent bioreporters have a range of applications from detection of bioavailable analytes including BTEX compounds, polyaromatic hydrocarbons, metals and other environmental pollutants^23^. However, most previous bacterial bioluminescence applications were for water and soil-toxicity monitoring and based solely on bioluminescence reduction. In short, these assays use naturally luminescent bacteria such as *Vibrio fischeri* and correlate the toxicity level with the reduction of light intensity. As can be expected with these types of inverse assays (toxic analyte increase results in signal decrease), many factors other than the toxicity level can also contribute to signal reduction. The organism used for bioluminescent testing in this report *P. fluorescens* M3A generates photons by enzymatic activity using the same system found in *Vibrio fischeri*. Therefore, the luminescent signal is dependent upon oxygen diffusion and nutrients levels. When measurements were conducted in a glass tube, initial vortexing generated the brightest light level by increasing available oxygen, which gradually decreased within 2–3 minutes. Therefore, the NREA algorithm was applied to the first three to five consecutive images, which required 45–180 seconds total, thus ensuring that the measurement was performed for the highest level of photon generation within the dynamic changes. However, it is important to note that *P. fluorescens* M3A is not the brightest organism used for whole cell bioluminescent assays or represents an equal surrogate for enzymatic assays with luminescent output. In past research using luminescence, results are always relative and reported within the limitations of both the organism (light levels) and the detector (sensitivity). Recent developments in enzymatic light production primarily the luciferase (Nanoluc) from *Oplophorus gracilirostris* which shows increased signal strength when compared to both the firefly and bacterial luminescent systems^24^. Increased signal strength also allows the use of fewer cells reducing oxygen limitations. Therefore, the system presented here will only increase in its applicability as luminescent assays are improved.

## Material and Methods

### Theoretical signal-to-noise ratio

In detection systems, it is critical to ensure the best signal-to-noise ratio (SNR). To estimate the theoretical SNR of the proposed measurement modality, SNR models were calculated for three detectors (silicon photodiode (PD), avalanche photodiode (APD), and photomultiplier tube (PMT)) when incident optical power ranges from 1 μW to 1 fW. Based on several previous reports on measurement systems^25^,^26^,^27^, total noise of the system can be formulated as the contribution from shot noise (photon generated and dark current):


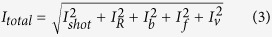


where *I*_shot_ is the shot noise from both dark current and photon signal current, *I*_R_ is the Johnson noise, and *I*_f_ and *I*_v_ are related to amplifier noise. Detailed derivation and simulation parameters are provided in the supplementary section. Therefore, actual SNR can be formulated as


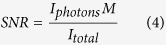


where *I*_photons_ is photon-generated current, *M* is the amplification ratio, which is typically ~60 for an APD and ~10^6^ for a PMT. Based on these assumptions, estimated SNRs for three detector families are shown in Figure S1. When there is a sufficient amount of light, contribution from all other noise is smaller than shot noise and there is not much difference in SNR among the detectors. However, when the incident power sinks below 10 nW, the SNR of a photodiode deteriorates much faster than that of its counterparts. Even with this model estimation, CMOS sensors, which fundamentally operate based on the photodiode principle, can detect nano- to picowatt (10^−10^–10^−12^ W) levels of incident photon signal. Based on this estimated SNR, we explored the limit of detection of the commercial smartphone camera on bacterial bioluminescence detection.

### Detector chamber design

A smartphone cradle was designed with two functions, one as a smartphone holder and the other as a detector chamber. The cradle was 3D printed utilizing a commercial printer (Replicator 2X 3D printer, MakerBot Industries, Inc., New York, NY, USA) with black acrylonitrile-butadiene-styrene (ABS) (Fig. 3). A smartphone holder was designed for each of two smartphone models (Oneplus One and iPhone 5 S (see Fig. 3(A,B)) since the physical dimension of the handsets and camera locations were different while the detector chamber was designed as inter-changeable chamber to be inserted into either of the tested smartphone holders. The design guarantees a light-tight access and consistent lateral (XY) and vertical (Z) locations for measurement across different smartphone brands. The detector chamber includes a removable plano-convex lens (PCX) (diameter 10 mm, focus 25 mm, Edmund optics #63–487) and an exchangeable inner case. To enhance the detection efficiency in low light situations, three different types of material, default ABS, a reflector polymer film (R-MG98-0810-AD00-N-D02), and an optical mirror (Edmund Optics first surface mirror 4–6λ), were integrated into three separate inner cases (see Fig. 3(C–E)). The effect of both lens and inner surface material were measured and analyzed.

### Formulation of the noise-minimization algorithm

**Figure 7:**
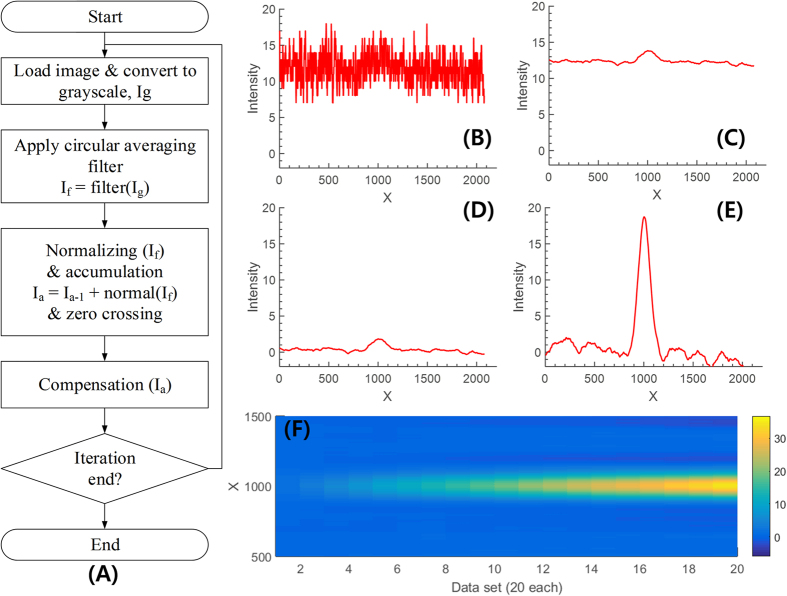
Flow chart and compensated signal at each step of the NREA (**N**oise reduction by Ensemble Averaging) algorithm. To verify and quantify the effect of the algorithm, a green LED (light intensity measured 2.08 μW at 60 mm from the camera, using a commercial power meter) with ND filter (OD 5.133) is used as a light source. The light intensity in front of the camera after the ND filter is computed as 20 pW. (**A**) Flow chart of the algorithm and cross-section near the center of the LED (center x axis: 1011 pixel) on the tested sample image sets for (**B**) gray-scaled image Ig, (**C**) after applying a circular averaging filter, (**D**) compensation done (slant, curve, zero crossing, and intensity level normalized) of a single image, and (**E**) after accumulation of ten images. (**F**) Progressive accumulation result of 20 images using NREA, which effectively enhances the signal without increasing the noise level.

Since the light intensity of the bioluminescence signal from the sample is very weak (in the range of tens of nW to 1 pW) and the sensitivity of the smartphone camera is limited (up to ISO 1600), a long exposure time or an equivalent technique is required to capture the signal. As the Android software development kits (SDKs) did not allow users to control manual exposure time prior to the latest version 5.0 (Lollipop)^28^, we captured multiple images (up to 40) of the same sample and numerically accumulate them to have the equivalent of a long-exposure camera. Utilizing the accumulation, we can capture a very weak signal. However, noise level simultaneously increases, which does not significantly improve SNR. To reduce the noise and effectively enhance the signal level, we developed an algorithm called noise reduction by ensemble averaging (NREA). Figure 7(A) shows the flow chart of the algorithm and the compensated signal of each step. The measured raw image from the smartphone camera is modeled as


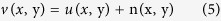


where v(x, y), u(x, y), and n(x, y) stand for measured image, true image, and noise perturbation, respectively, and they are a matrix form. The noise-perturbation term for a low-light environment is assumed to be a Gaussian distribution^29^. To quantify the amount of the noise from the measured image, the signal-to-noise ratio on a logarithmic decibel scale is defined as


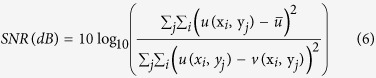


where 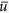 is an average of the true image. 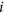 and 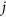 stands for 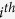 and 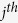 pixel on the image for horizontal and vertical direction. Figure 7(B) shows the cross section of the measured raw image near the center imaging area. As the model (Eq. 5) depicts, the measured image consists of a relatively low frequency true signal and a high frequency noise signal with an offset. To reduce the noise, a circular averaging filter, worked as low-pass filter (LPF), is applied (Fig. 7(C)). The goal of the NREA algorithm is to minimize the intensity value in a background area, while accumulating the true signals only within the region of interest. Figure 7(D) shows a cross-section of result after normalization and zero crossing technique is applied to the raw image. Figure 7(E) shows the accumulation result of 10 images using the NREA algorithm, while Fig. 7(F) shows the progressive accumulation result of 20 images using the NREA algorithm. Figure S2(A) shows the definition of the parameters for NREA. The show line and the extracted point stand for an automatically selected cross-sectional line and its maximum for the analysis. The sum area stands for the area for area analysis. The zero-crossing line is considered as a no-signal area, so the area should be kept as zero in theory. The mean value of the zero-crossing line is used to compensate the offset of the accumulation by NREA.

### Radiant flux calibration

Before measuring the actual bioluminescence, we tested the detection limit of the smartphone cameras utilizing the proposed detection chamber and NREA algorithm. To provide a stable and constant light in a similar spectral region, a green LED (C566C-GFS-CV0Z0792, Cree, Inc., Durham, NC, USA) with 2.08 μW light intensity was positioned 60 mm away from the smartphone camera. In addition, we placed a set of neutral-density filters (absorptive type) in front of the green LED to artificially generate a range of light intensity from OD 4 to OD 8.

Table 1 shows the list of OD values versus their respective output optical power, which was measured by a commercial power meter (PD100D, Thorlabs, Newton, NJ, USA). For each OD, 20 images were captured, and the NREA algorithm from section 2.3 was applied to determine the final intensity from the camera image.

### Inter-phone comparison

Smartphones are manufactured with various proprietary aspects even for the same optical and imaging components. Thus, we have incorporated a standard test for five different handsets (four Android and one iOS smartphone) and compared their performance in artificially generated low-light level condition. First, we fixed two of three major camera parameters (ISO, aperture, and shutter speed) and compensated the third parameter for fair comparison. The same LED with filter OD5 was used as a model low-light signal. Then we compared the resulting output signal into 8-bit intensity levels. For the Android, the same app (Camera FV-5 2.79.2) was utilized to generate a series of images in the given condition. Therefore, only their internal algorithm and hardware specifications were reflected in their performance. Second, we explored the lowest possible light level that each smartphone camera could handle by utilizing their best low-light performance mode such as night mode and not restricting any parameters in the phone. We have tested from OD4 to OD8, which corresponds to a sub-pico Watt intensity for light at 500 nm.

### Sample preparation for bacterial bioluminescence

*P. fluorescens* M3A harbors a mini-Tn5 nahRGp-luxCDABE transposon in which the lux cassette originates from *Vibrio fischeri*. The nahRGp gene cassette consists of the lysR regulatory protein encoded by nahR and the sal promoter originating from plasmid NAH7 fused to the luxCDABE gene cassette^30^. The resulting construct results in a concentration dependent upregulation of the luxCDABE genes and resultant luminescence in the presence of salicylate. The salicylate bio-reporter *P. fluorescens* M3A^31^ was grown in 300-ml Erlenmeyer flasks containing 100 ml LB broth (in g/L: Tryptone 10, yeast extract 5, NaCl 10) with the antibiotic kanamycin (50 μg/mL) and the reporter analyte sodium salicylate (50 μg/mL). The culture was grown overnight in a shaking incubator at 25 °C and pH 7, until an optical density of 0.50 to 1.00 at 600 nm (OD600) was reached. To analyze different bioluminescence levels, dilutions of the initial culture in phosphate buffered saline were tested (0, 0.75, 0.5, 0.25, 0.1, 0.02, 0.04, 0.001). For each dilution, optical density, colony-forming units (CFUs), and bioluminescence were measured from triplicate samples for statistical comparison. In addition, to compare with the standard protocol, a SIRIUS luminometer (Berthold DetectionSsystem Gmbh, Pforzheim, Germany) was used for the reference output from the PMT platform.

## Conclusion

A smartphone-based bioluminescence detector called BAQS is proposed. The system utilizes both software (NREA algorithm) and hardware optimizations (sample chamber) to maximize photon-capture efficiency. The LED-based model system was calibrated against known input intensity controlled by a stack of ND filters and the effectiveness of the NREA algorithm and optical chamber were confirmed. *P. fluorescens* M3A was used for live bacterial bioluminescence and a detection limit of approximately 7.9 × 10^6^ CFU/ml was achieved by two representative Android and iOS smartphones with the developed sample chamber.

## Additional Information

**How to cite this article**: Kim, H. *et al*. Smartphone-based low light detection for bioluminescence application. *Sci. Rep.* **7**, 40203; doi: 10.1038/srep40203 (2017).

**Publisher's note:** Springer Nature remains neutral with regard to jurisdictional claims in published maps and institutional affiliations.

## Supplementary Material

Supplementary Information

## Figures and Tables

**Table 1:** Series of calibrated ND filter sets and respective OD values.

Set Number	ND filter combination	filter OD	P_in_ (pW)	cps (photon/s)
1	#2 + 4 + 5 + 7	4.099	165.6	4.17 × 10^8^
2	#7 + 8	4.607	51.41	1.29 × 10^8^
3	#6 + 7	5.009	20.37	5.12 × 10^7^
4	#1 + 5 + 6 + 7	5.510	6.43	1.62 × 10^7^
5	#1 + 5 + 6 + 8	6.089	1.69	4.25 × 10^6^
6	#1 + 4 + 6 + 8	6.581	0.556	1.39 × 10^6^
7	#1 + 3 + 6 + 8	7.030	0.194	4.90 × 10^5^
8	#6 + 7 + 8	7.602	0.052	1.32 × 10^5^
9	#5 + 6 + 7 + 8	7.976	0.022	5.68 × 10^4^

For number of photon calculation, λ = 500 nm was assumed with input power of 2.08 μW.
