# Supplementary material for: Smartphone-based low light detection for bioluminescence application
Source: Sci Rep. 2017 Jan 9;7:40203. doi: 10.1038/srep40203 (PMC5220360; doi:10.1038/srep40203)
Supplement: Supplementary Information [file srep40203-s1.doc]

Smartphone-based low light detection for bioluminescence application

Huisung Kim^¶§^, Youngkee Jung^¶§^, Iyll-Joon Doh^¶^, Roxana Andrea Lozano-Mahecha^Ψ^, Bruce Applegate^†¥^, and Euiwon Bae^¶*^

^¶^Applied Optics Laboratory, School of Mechanical Engineering, ^Ψ^Universidad Nacional Colombia-Palmira, Palmira Colombia, ^†^Department of Food Science, ^¥^Department of Biological Sciences, Purdue University, West Lafayette, Indiana 47907, USA

^*^Corresponding author:ebae@purdue.edu

^§^H. Kim and Y. Jung contributed equally to this work


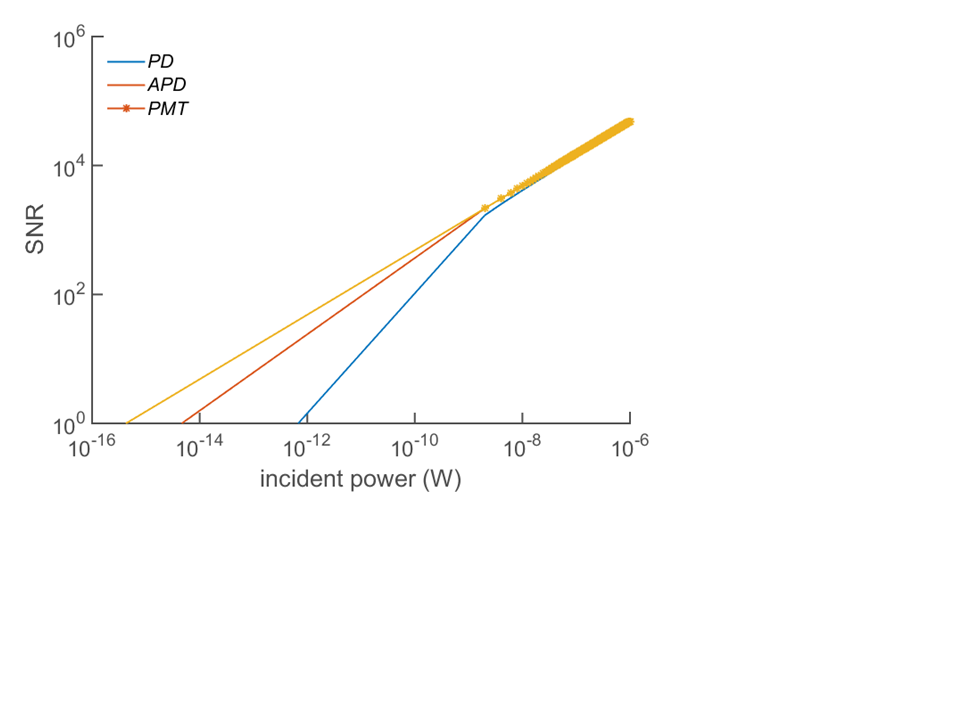


**Figure S1**. Estimation of the theoretical SNR for three different detectors (PD, APD, and PMT) for λ=500 nm, multiplication ratio (M) for set as 1, 30, and 10^6^ respectively. Beyond 10^-9^ W, all detectors are in shot noise limited region while on the extremely low light region, PMT and APD provides better sensitivity. For PDs, they still provide reasonable detection limit down to pW level of power.


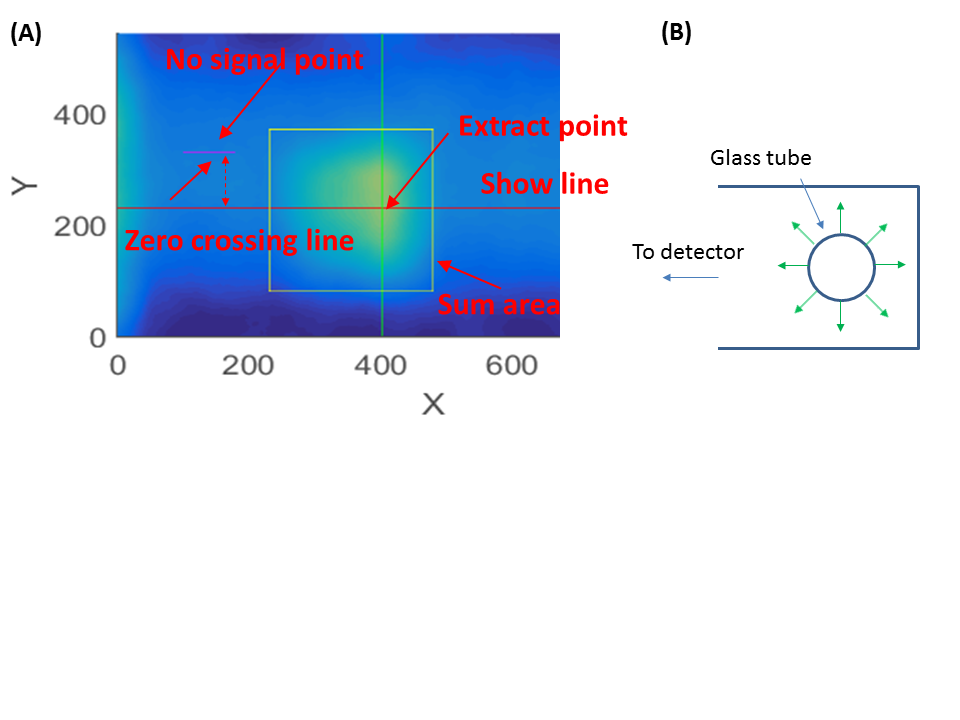


**Figure S2**. (A) Definition of the parameters for NEA. The show line and the extract point stands for automatically selected cross sectional line and maximum of the light intensity at the cross sectional line for the analysis respectively. The sum area stands for the area for area analysis. The zero crossing line is considered as no signal area, so the area should be kept minimum noise floor. (B) Layout of the sample chamber with respect to the smartphone camera. Only the photons that are within the receiving angle of the smartphone camera will be detected.


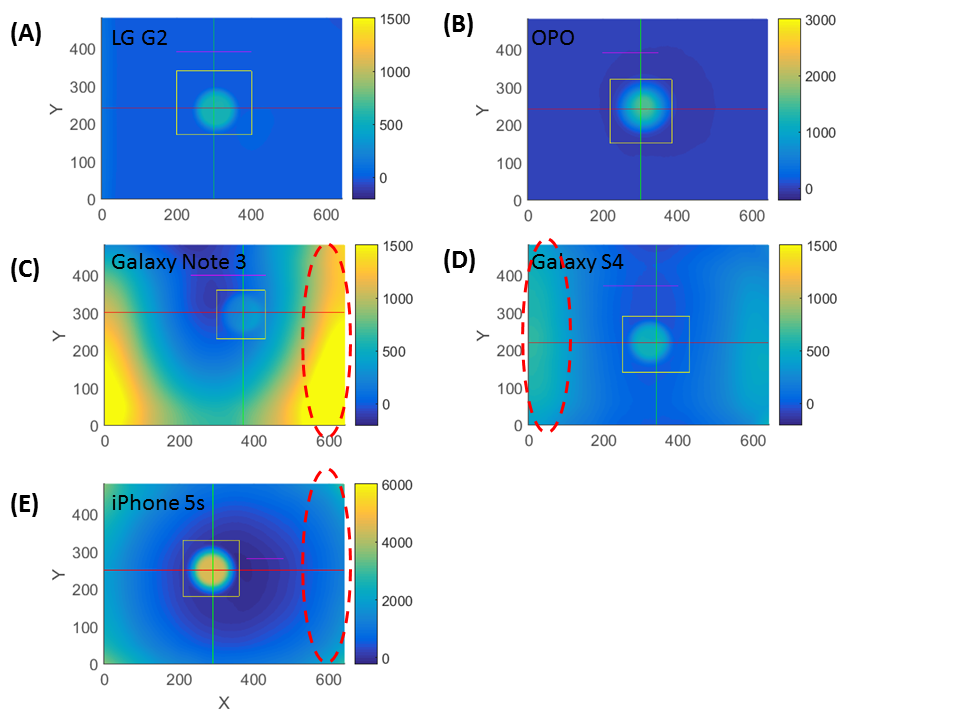


Figure S3. Example of leakage phenomena from CMOS sensors of different phone types. Current setting is given as OD 5 with FV-5 app and standard condition. Except for (A) LG G2, (B) OnePlue One, rest of the handsets ((C) Galaxy Note 3, (D) Galaxy S4, (E) iPhone 5S) generated an inherent background signal level similar to the desired bioluminescence signal (marked by red-dotted area).


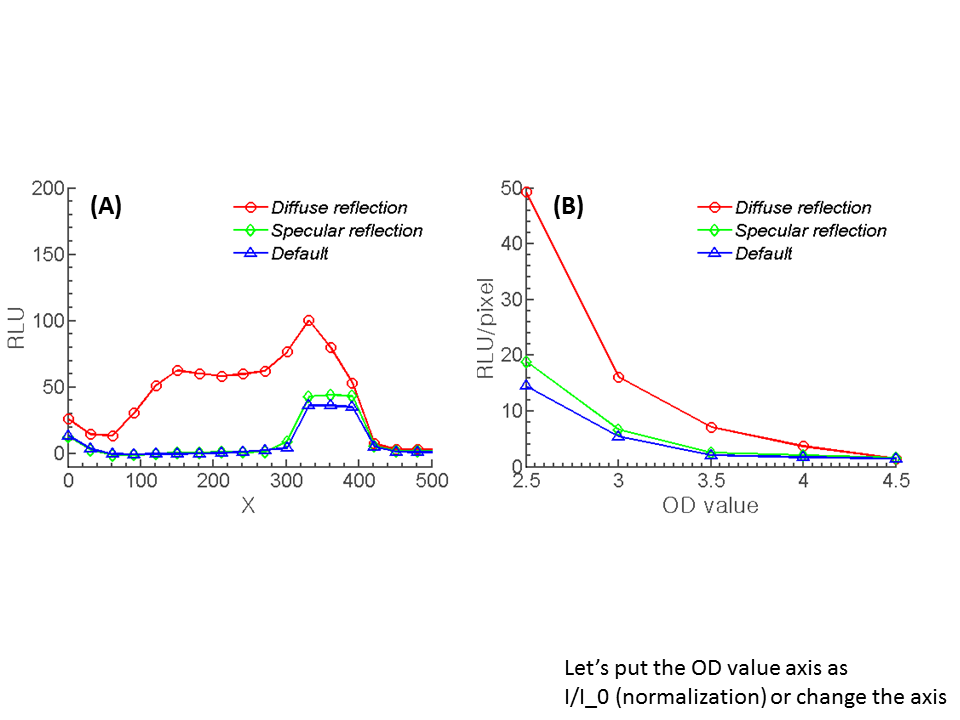


Figure S4. Effect of the optical chamber design. (A) Comparison of three different chambers: diffusive reflection film, first surface optical mirror, and default ABS surface. 1-D cross-sectional image shows the effect of enhanced photon captures by the reflection film method. (B) Efficacy of the three method versus different input powers.


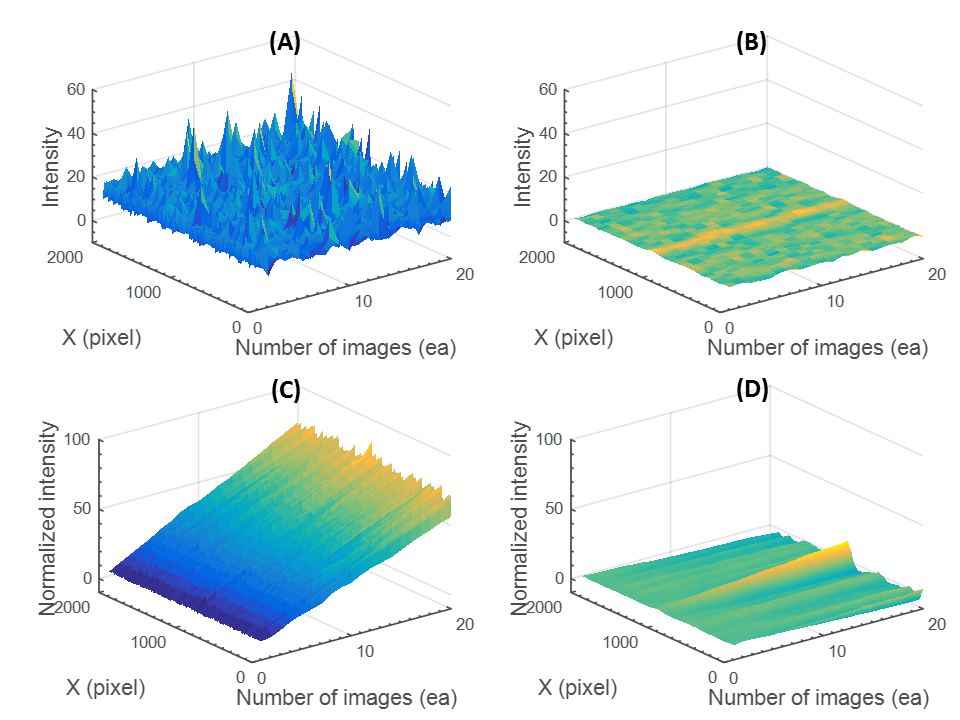


**Figure S5**. Comparison of normal (no algorithm applied) and NREA algorithm applied accumulation result. Cross section of 20 images near the center area (center of LED area of each image, x: 1011) for the (A) raw signal, and (B) the NREA algorithm applied signal. Because of the noise, the LED area is not recognizable at raw signal, while, NREA algorithm applied case shows relatively clear signal of the LED comparing to the background noise. The accumulation result of each image for (C) w/o the algorithm, and (D) NREA algorithm applied case. Even the simple accumulation result shows higher absolute intensity value at the signal area, the noise level is also high. Meanwhile, NEA algorithm case shows better contrast between background and signal area. Another advantage of applying the NREA is non-signal area values is kept approximately zero, which is helpful for better contrast.


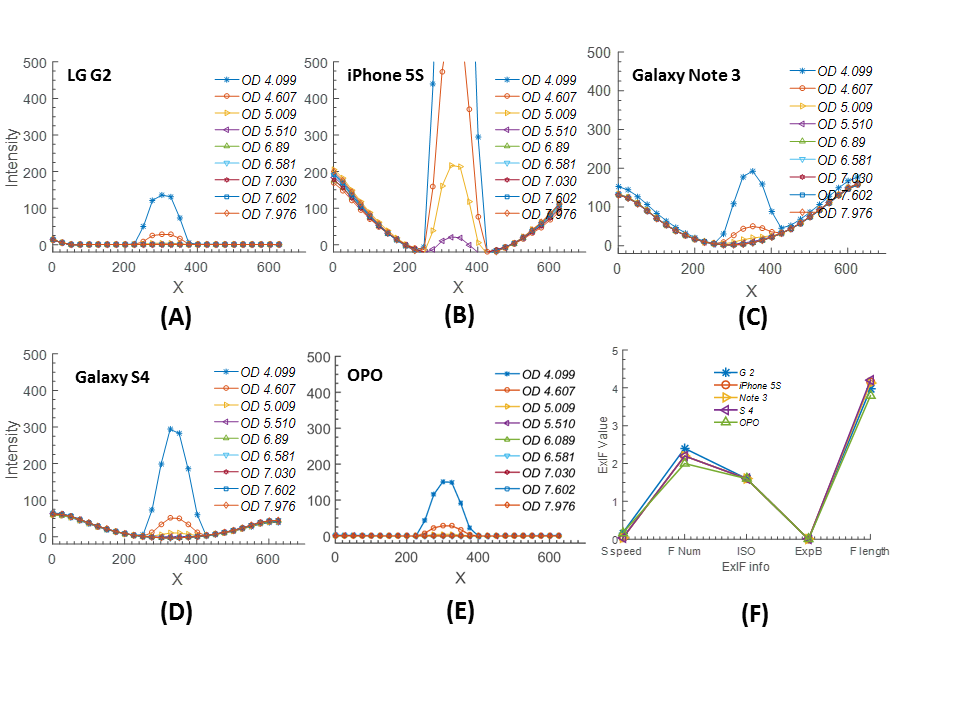


**Figure S6**. Inter-phone comparison of five different brand of smartphones. With identical experimental condition (f=25mm plano-convex lens and 2.08 uW of power) utilizing the same app (except for iPhone 5S), OD 4.5 was the minimum detectable intensity level for LG G2 and OPO and OD5 for Galaxy Note 3 and S4 and OD5.5 for iPhone 5S.


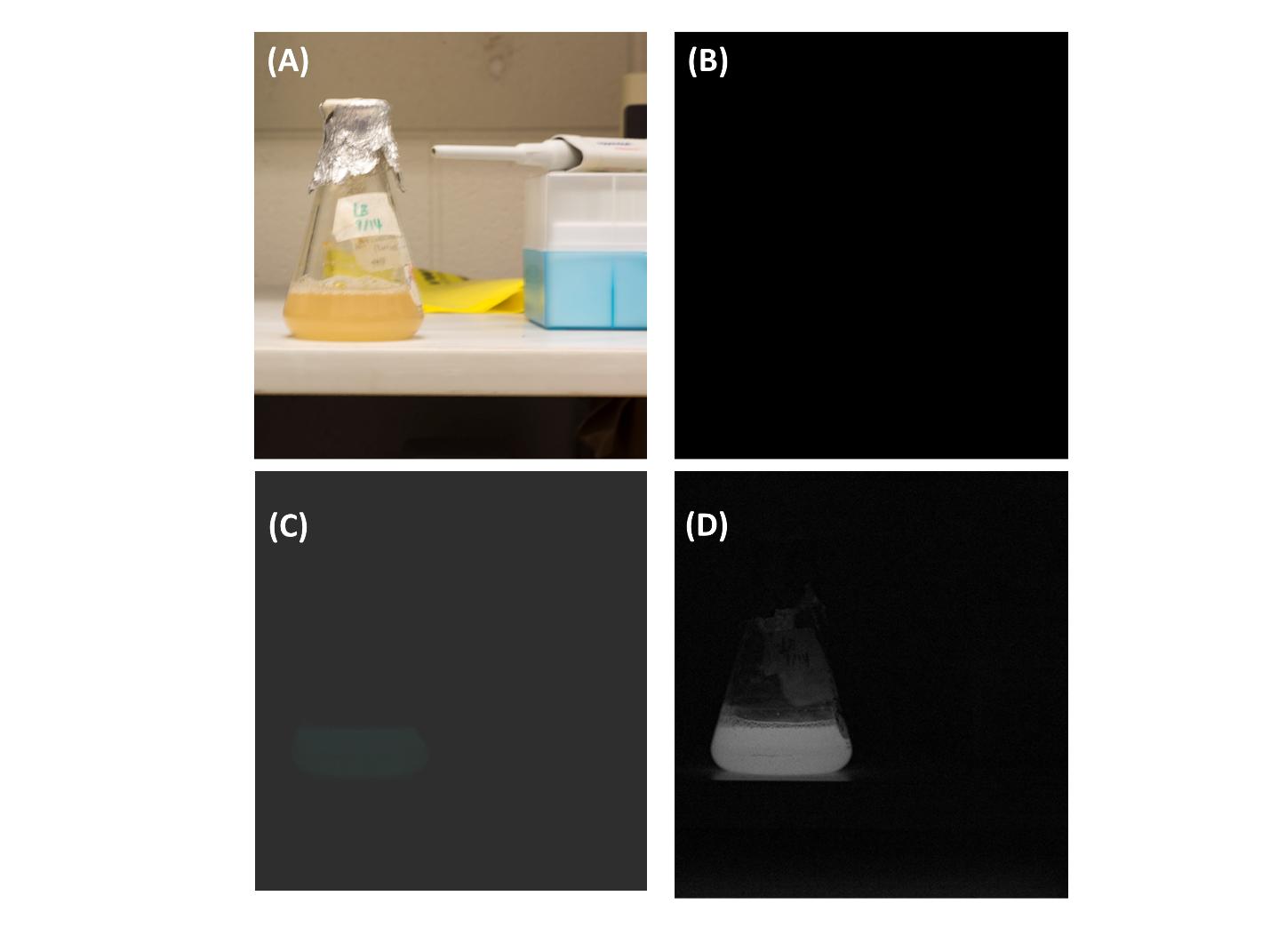


**Figure S7**. Comparison of the standard camera image and NREA algorithm. (A) No dilution (7.8 x 10 ^8 CFU/ml) with fluorescent light (Nikon D810, f1.8, 1/3, ISO 1000), (B) without light (Nikon D810, f1.8, 1/3, ISO 1000), (C) (Nikon D810, f1.8, 1”, ISO 12800-brightness was adjusted to +20 for better visibility), (D) NREA with 10 images processing.
